# The role of social support and self-control in tobacco consumption: a cross-sectional study among tobacco consumers and non-consumers

**DOI:** 10.1186/s40359-023-01226-y

**Published:** 2023-06-29

**Authors:** Atefeh Homayuni, Zahra Hosseini

**Affiliations:** 1 Student Research Committee, Faculty of Health, Hormozgan University of Medical Sciences, Bandar Abbas, Iran; 2 Tobacco and Health Research Center, Hormozgan University of Medical Sciences, Bandar Abbas, Iran

**Keywords:** Perceived social support, Self-control, Tobacco, Cigarette, Hookah

## Abstract

**Introduction:**

Tobacco use is recognized as one of the most important causes of preventable death due to non-communicable diseases and disability worldwide. The present study was conducted with the aim of comparing social support and self-control between tobacco consumers and non-consumers in Hormozgan Province.

**Methods:**

The present cross-sectional study was conducted on the adult population above the age of 15 years living in Hormozgan province. A total number of 1,631 subjects were selected using a convenient sampling method. An online questionnaire was used to collect the data, which consisted of three sections: demographic information, Zimet’s perceived social support and Tangney’s self-control questionnaires. In the present study, Cronbach’s alpha coefficients of social support and self-control questionnaires were 0.886 and 0.721, respectively. Data were analyzed using chi-squared test, Mann-Whitney U-test, and logistic regression analysis with SPSS software (v. 25).

**Results:**

Among the participants, 842 (51.6%) reported to be tobacco non-consumers, and 789 (48.4%) reported to be consumers. The mean scores of perceived social support among the consumers and non-consumers were 4.6 ± 1.012 and 4.93 ± 0.518, respectively. The mean scores of self-control among the consumers and non-consumers were 2.74 ± 0.356 and 2.75 ± 0.354, respectively. There was a significant difference among tobacco consumers and non-consumers in gender, age, education level and job status (*p* < 0.001). The results showed that the mean scores of social support, support received from family and others were significantly higher in non-consumers than in consumers (*p* < 0.001). There was no statistically significant difference between the mean scores of self-control, self-discipline, and impulse control in consumers and non-consumers (*p* > 0.05).

**Conclusion:**

According to our findings, tobacco consumers received more social support from family and others compared to non-consumers. Considering the important role of perceived support in tobacco consumption, this variable should receive copious attention in developing interventions and trainings, especially family education workshops.

**Supplementary Information:**

The online version contains supplementary material available at 10.1186/s40359-023-01226-y.

## Introduction

Tobacco consumption is the first preventable risk factor for chronic diseases, premature death and disability worldwide [1]. More than 8 million people died in 2019 due to tobacco-related diseases [2]. As anticipated, the mortality rate induced by smoking will increase to 8.3 million by 2030, which is the largest growth in low- and middle-income countries [3]. Diseases attributed to smoking include lung and heart diseases, chronic respiratory diseases, cancers, diabetes, autoimmune diseases and osteoporosis [4]. In addition to health outcomes, economic damages due to lowered productivity and increased health care costs are also known as the negative effects of tobacco consumption [5].

The number of smokers worldwide is approximately 1.3 billion, and over 80% of the world’s 1.3 billion tobacco users live in low-and middle-income countries. In 2020, 22.3% of the global population used tobacco, 36.7% of all men and 7.8% of the world’s women [6, 7]. The prevalence of current tobacco use in Iran was 25.2% (24.4–25.9) in men versus 4% (3.7–4.3) in women and this rate was higher in rural areas among second wealth group [8]. The results of a study conducted by Varmaghani et al. [9] in 2016 showed that more than 15% of the Iranian population were currently smokers for all types of tobacco and a little less than one of ten Iranians were current cigarette smokers. The prevalence of current tobacco smoking among adult men and women at the time of study was 24.4% and 3.8%, respectively. In this survey, the highest prevalence rates of current cigarette smoking were observed in West Azerbaijan with 14%, 13.7% in Markazi, and the lowest prevalence rates were found to be 4.7% in North Khorasan and 5% in Golestan.

Studies showed that several factors such as personal inefficiency, family inefficiency, a vulnerable social environment, the context of consumption, the need for effective supervision, beliefs of being useful physically and psychologically are important causes of smoking and smokeless tobacco initiation and continuation [10–12]. Moreover, misconceptions about the safe use, the availability and low cost of hookah are among the reasons for the high prevalence of hookah consumption [13].

Review literature showed that the perceived social support plays an important role as a coping mechanism against high-risk behaviors such as smoking, alcohol/drug abuse, and high-risk sexual behaviors [14]. Social support refers to supportive behaviors and sources of social bonds that include emotional support, intimacy, positive interaction, and financial support [15]. Social support as an emotional coping strategy can protect people by preventing the occurrence of stressful conditions and can help them to evaluate stressful events in a less threatening way [16]. Social support can be examined at three levels: family, friends and important others [17].

Among the other variables that can affect the tendency towards tobacco consumption is self-control. Self-control was defined by Mayer and Salovey (2003) as the correct use of emotions. They believed that the ability to regulate emotions increases one’s ability to relieve oneself, understand anxiety, depression or common cases of boredom. Those enjoying an intrinsic self-control believe that success or failure depends on their own efforts or abilities, but people with extrinsic self-control believe that other factors such as luck or task difficulty are translated into success or failure [18]. According to the general theory of crime, individual differences in self-control are related to some behaviors such as alcohol consumption, smoking in young people, marital instability and accidents in adulthood. This theory highlights the relationship between low self-control and the ability to commit criminal and risky behaviors [19]. Daly et al. [20] pointed out the relationship between self-control and smoking and showed that participants with high self-control showed lower rates of smoking across the seven year period of the study.

According to what was mentioned above, it can be said that personality traits and perceived social support may interact in predicting tobacco consumption. In Iran, these predictive factors have been studied rarely. Furthermore, due to the growing rate of cigarette smoking and hookah consumption and their negative consequences, recognizing the factors and motivators that contribute to tobacco consumption will play a key role in decision making and planning to prevent it. Thus, in the present study we will compare social support and self-control among tobacco consumers and non-consumers in Hormozgan Province.

## Materials and methods

### Study Design

This study was based on a descriptive-correlational design. The statistical population of this study consisted of the general population over 15 years old in Hormozgan Province (in southern Iran).

### Sample size and sampling procedure

According to the previous study [21] the prevalence of smoking in Hormozgan province was 20%.Sample size was calculated using Cochran’s formula:

$$n=\frac{\left(Z_{1-{\displaystyle\frac a2}}\right)^2\times\;pq}{d^2}$$

*N* = the minimum required sample size, z = level of confidence (1.96), *p* = parameter for sample calculation, d = margin of error (0.05).

Based on this formula, a sample size of 1536 anticipated for the study. Considering that in this study, a convenient sampling was used, the sample size was increased to 1600.

The inclusion criteria for the sample selection included: access to the Internet to answer questions, literacy, willingness to participate in the study, and the minimum age of 15 years. Exclusion criteria were unwillingness to participate in the study and incomplete answers to the main questions of the questionnaires.

Online questionnaires were used to collect data. We made the link of the questionnaire available to the participants through health care providers and health center assistant nurses in the cities in the relevant channels (Telegram, WhatsApp, Instagram, E-Gap). If someone was interested to participate in the study, they needed to complete an informed consent form and then answer to the questions. The samples that received the link were asked to send it to other people they know. On the first page of the questionnaire, the objectives of the study were clearly explained to the participants and they were reminded that mentioning their first and last names would not be necessary and their information would be kept confidential.

### Data collection tools

Data were collected using the demographic information questionnaire, self-control questionnaire and perceived social support scale.

#### Demographic information questionnaire

This questionnaire was developed for this study. The assessed demographic characteristics were gender, age, education level, job status and marital status.

#### Self-control questionnaire

This questionnaire is an 13-item self-report scale, which developed by Tangney et al. [22] and measure the level of respondents’ control over themselves. The responses are scored on a 5-point Likert scale ranging from 1 (not at all) to 5 (very much). Questions 2, 3, 4, 5, 7, 9, 10, 12 and 13 were reversely scored. Overall scores ranged from 13 to 65 points whereby the higher score indicated a higher self-control. The validity and reliability of this questionnaire were estimated and confirmed in a study conducted by Mousavimoghadam et al. [23]. Tangney et al. [22] confirmed its validity through estimating its correlation with scales of academic achievement, adaptability, positive relationships, and interpersonal skills. Cronbach’s alpha coefficients for this scale in two statistical populations were 0.83 and 0.85. In the present study, Cronbach’s alpha coefficient was 0.721.

#### Perceived social support scale

This scale was developed by Zimet et al. [17] with 12 items to measure the level of perceived social support from three sources: family, friends and important others. Each question is scored on a 7-point Likert scale (1: completely disagree to 7: completely agree). A higher score would indicate a higher perceived social support. Cronbach’s alpha coefficient for this scale was reported 0.89 [17]. Rostami et al. [24] reported the Cronbach’s alpha coefficients for each of the subscales between 0.76 and 0.89. In the present study, Cronbach’s alpha coefficient was 0.886.

### Data analysis

Data were analyzed using SPSS version 25 statistical software. Mean, standard deviation (SD), minimum and maximum scores were used to report interval variables and frequency and percentage were reported to categorical variables. The Chi-squared test was used for testing the difference between consumers and non-consumers in terms of demographic variables. Mann-Whitney U-test (due to the non-normality of the data) was used to test the difference between the mean scores of the two groups according to social support and self-control variables. Logistic regression analysis was run to test the relationship between demographic variables and tobacco consumption status, as well as the relationship between social support and self-control with tobacco consumption status. The level of significance was considered to be 95% (*p* < 0.05).

### Ethical considerations

This study was approved by the Ethics Committee of Hormozgan University of Medical Sciences (#IR.HUMS.REC.1401.232). Participants were assured that their information would remain confidential. Written informed consent was obtained from all participants and from legal guardians for the participants who were below 16 years of old.

## Results

### Participants’ characteristics

A total number of 1631 questionnaires were completed and returned. Among them, 842 participants (51.6%) were tobacco non-consumers and 789 (48.4%) were consumers. The participants’ demographic information is shown in Table 1.

**Table 1:** Participants’ demographic information in consuming vs. non-consuming groups and in general

	Tobacco consumers		Tobacco non-consumers		Total	
	Frequency	Percent	Frequency	Percent	Frequency	Percent
**Gender**						
Female	194	24.6	461	54.8	655	40.2
Male	595	75.4	381	45.2	976	59.8
**Age**						
15–28 years	102	12.9	123	14.6	225	13.8
29–39 years	111	14.1	366	43.5	477	29.2
40–49 years	311	39.4	251	29.8	562	34.5
50–80 years	265	33.6	102	12.1	367	22.5
**Education level**						
Under diploma	482	61.1	284	33.7	766	47
Associate degree	164	20.8	197	23.4	361	22.1
Bachelor’s degree	107	13.6	201	23.9	308	18.9
Master’s degree and higher	36	4.6	160	19.0	196	12.0
**Job status**						
University student	37	4.7	110	13.1	147	9.0
Unemployed	44	5.6	14	1.7	58	3.6
Housewife	94	11.9	237	28.1	331	20.3
Employee	94	11.9	329	39.1	423	25.9
Self-employed	520	65.9	152	18.1	672	41.2
**Marital status**						
Single	133	16.9	123	14.6	256	15.7
Married	656	83.1	719	85.4	1375	84.3

### Descriptive statistics

Descriptive statistics (mean, standard deviation, minimum and maximum values) of research variables are reported in Table 2.

**Table 2:** Descriptive statistics of research variables

Tobacco consuming status		Minimum	Maximum	Mean ± SD
**Tobacco consumers**	Social support	1.00	7.00	4.60 ± 1.012
	Family support	1.00	7.00	5.10 ± 1.223
	Friends support	1.00	7.00	3.77 ± 1.213
	Others support	1.00	7.00	4.94 ± 1.252
	Self-control	1.67	4.00	2.74 ± 0.356
	Self-discipline	2.00	4.63	3.28 ± 0.422
	Impulse control	1.00	3.50	1.67 ± 0.645
**Tobacco non-consumers**	Social support	1.00	7.00	4.93 ± 0.518
	Family support	1.00	7.00	5.59 ± 0.606
	Friends support	1.00	7.00	3.71 ± 0.981
	Others support	1.00	7.00	5.48 ± 0.633
	Self-control	1.67	4.00	2.75 ± 0.354
	Self-discipline	1.88	4.50	3.29 ± 0.439
	Impulse control	1.00	3.25	1.67 ± 0.616

As the results showed, the mean and standard deviation scores of research variables for tobacco consumers and tobacco non-consumers were as follows: social support (4.60 ± 1.012, 4.93 ± 0.518), family support (5.10 ± 1.223, 5.59 ± 0.606), friends support (3.77 ± 1.213, 3.71 ± 0.981), others support (4.94 ± 1.252, 5.48 ± 0.633), self-control (2.74 ± 0.356, 2.75 ± 0.354), self-discipline (3.28 ± 0.422, 3.29 ± 0.439) and impulse control (1.67 ± 0.645, 1.67 ± 0.616).

### Chi-squared test results

Table 3 showed the results of the chi-squared test to investigate the differences between consumers and non-consumers according to demographic variables (gender, age, education level, job status and marital status). The results showed that in terms of gender, the number of female non-consumers was significantly higher than female consumers and vice versa. The number of male consumers was significantly higher than the number of male non-consumers (*p* < 0.001). As for age, the results showed that in the 15-28-year age group, the number of consumers and non-consumers was the same; thus, there was no statistically significant difference between these groups (*p* > 0.05). In the 29-39-year age group, the number of non- consumers was significantly more than consumers. Yet, in the 40-49-year age group (*p* < 0.05) and 50-80-year age group (*p* < 0.001), the number of consumers was significantly higher than non-consumers. As for the education level, in the subcategory “under diploma”, the number of consumers was significantly higher than the number of non-consumers (*p* < 0.001). In the subcategory “Associate degree”, the number of consumers and non- consumers was the same and, thus, there was no statistically significant difference (*p* > 0.05). In the “Bachelor’s degree” and “Master’s degree and higher” subcategories, the number of non-consumers was significantly higher than consumers (*p* < 0.001). Concerning the job status, in the unemployed and self-employed, the number of consumers was significantly higher than the number of non- consumers (*p* < 0.001). In students, housewives and employees, the number of non- consumers was significantly higher than consumers (*p* < 0.001). And finally, in relation to the marital status, in the single and married participants, the number of consumers and non- consumers was the same and no statistically significant difference was found (*p* > 0.05).

**Table 3:** Differences between tobacco consumers and non-consumers in terms of demographic variables

	Tobacco consumers		Tobacco non-consumers		Result	
	Observed N	Expected N	Observed N	Expected N	Chi-Squared	sig
**Gender**						
Female	194	327.5	461	327.5	108.838	0.000^**^
Male	595	488.0	381	488.0	46.922	0.000^**^
**Age**						
15–28 years	102	112.5	123	112.5	1.960	0.162
29–39 years	111	238.5	366	238.5	136.321	0.000^**^
40–49 years	311	281.0	251	281.0	6.406	0.011^*^
50–80 years	265	183.5	102	183.5	72.395	0.000^**^
**Education level**						
Illiterate	21	21.0	0	0.0	-	-
Under diploma	461	372.5	284	372.5	42.052	0.000^**^
Associate degree	164	180.5	197	180.5	3.017	0.082
Bachelor’s degree	107	154.0	201	154.0	28.688	0.000^**^
Master’s degree and higher	36	98.0	160	98.0	78.449	0.000^**^
**Job status**						
University student	37	73.5	110	73.5	36.252	0.000^**^
Unemployed	44	29.0	14	29.0	15.517	0.000^**^
Housewife	94	165.5	237	165.5	61.779	0.000^**^
Employee	94	211.5	329	211.5	130.556	0.000^**^
Self-employed	520	336.0	152	336.0	201.524	0.000^**^
**Marital status**						
Single	133	128.0	123	128.0	0.391	0.532
Married	656	687.5	719	687.5	2.887	0.089

**p<0.01, *p<0.05

### Mann-Whitney U-test results

Table 4 showed the results of Mann-Whitney U-test to investigate the differences between tobacco consumers and non-consumers in terms of perceived social support and self-control and their subscales. According to the results, the mean scores of social support, the support by family and important others in the non- consumers group were significantly higher than the consumers group (*p* < 0.001). but the mean score of support received by friends did not differ significantly between consumers and non- consumers (*p* > 0.05). Concerning self-control and its subscales, the results revealed no statistically significant differences between the mean scores of self-control, self-discipline and impulse control in consumers and non- consumers (*p* > 0.05).

**Table 4:** Social support, self-control and their subscales in tobacco consumers and non-consumers

		N	Mean ± SD	Mann-Whitney U	Sig.
Social support*	Tobacco consumers	789	4.60 ± 1.012	265941.500	0.000^**^
	Tobacco non-consumers	842	4.93 ± 0.518		
Family support*	Tobacco consumers	789	5.10 ± 1.223	261519.000	0.000^**^
	Tobacco non-consumers	842	5.59 ± 0.606		
Friends support*	Tobacco consumers	789	3.77 ± 1.213	329159.000	0.751
	Tobacco non-consumers	842	3.71 ± 0.981		
Others support*	Tobacco consumers	789	4.94 ± 1.252	244851.000	0.000^**^
	Tobacco non-consumers	842	5.48 ± 0.633		
Self-control*	Tobacco consumers	789	2.74 ± 0.356	326984.000	0.584
	Tobacco non-consumers	842	2.75 ± 0.354		
Self-discipline*	Tobacco consumers	789	3.28 ± 0.422	325905.500	0.507
	Tobacco non-consumers	842	3.29 ± 0.439		
Impulse control*	Tobacco consumers	789	1.67 ± 0.645	330985.000	0.890
	Tobacco non-consumers	842	1.67 ± 0.616		

**p<0.01

### Correlation between demographic variables and tobacco consumption status

#### Hypothesis 1. There is a statistically significant relationship between demographic variables and tobacco consumption status

Logistic regression analysis was used to test this research hypothesis as the variable was bimodal (tobacco consuming vs. non-consuming). The findings summarized in Table 5 showed that when the demographic variables (age, gender, education, job status, marital status) are added to the model, out of 842 non-consumers, 681 were appropriately classified as non- consumers and 161 as consumers. Therefore, 80.9% of the predictive power of the model was accurate (identification). Out of 789 consumers, 206 were appropriately classified as non- consumers and 583 as consumers. In other words, 73.9% was accurately predicted (sensitivity), and the overall accuracy of the model was 77.5%.

**Table 5:** Classification table

Step 0					Step 1		
Observed		Predicted			Predicted		
		Tobacco consumption		Percentage Correct	Tobacco consumption		Percentage Correct
		Non-consuming	consuming		Non-consuming	Consuming	
Tobacco consumption	Non-consuming	842	0	100.0	681	161	80.9
	Consuming	789	0	0.0	206	583	73.9
Overall Percentage				51.6			77.5

As shown in Table 6, in block 1, the significance level of the fixed effect variable is greater than 0.05 and the rest of the variables (demographic variables) need to be present in the model. As the non-consuming code is 0 and the consuming code is 1, the prediction rate of education level and marital status variables is lower than 1. Therefore, the variables of education level and marital status are effective.

**Table 6:** Variables in the equation

		B	S.E.	Wald	Df	Sig.	Exp (B)
**Step 1**	Gender	0.548	0.146	14.179	1	0.000^**^	1.730
	Age	0.501	0.083	36.889	1	0.000^**^	1.651
	Education level	− 0.551	0.063	77.742	1	0.000^**^	0.576
	Job status	0.723	0.076	89.411	1	0.000^**^	2.061
	Marital status	-2.936	0.265	122.534	1	0.000^**^	0.053
	Constant	0.083	0.286	0.085	1	0.771	1.087

**p<0.01

### Correlation between self-control and social support with tobacco consumption status

#### Hypothesis 2. There is a statistically significant relationship between self-control and social support with tobacco consumption status

The results of Table 7 indicated that when self-control and social support variables were included in the model, out of 842 participants who did not consumed tobacco, 627 were accurately classified as non-smokers and 215 were assigned to the non-smokers class. They belong to the consumer class. Therefore, 74.5% of the model prediction is accurate (identification). Out of 789 participants who consumed, 447 were in the non-consumer group and 342 in the consumer group, assuming that they consumed tobacco. 43.3% was accurately predicted (sensitivity) and the total accuracy of the model was 59.4%.

**Table 7:** Classification table

Step 0					Step 1		
Observed		Predicted			Predicted		
		Tobacco consumption		Percentage Correct	Tobacco consumption		Percentage Correct
		Non-consuming	consuming		Non-consuming	Consuming	
Tobacco consumption	Non-consuming	842	0	100.0	627	215	74.5
	Consuming	789	0	0.0	447	342	43.3
Overall Percentage				51.6			59.4

As indicated in Table 8, in block 1, the significance level of social support and the fixed effect is lower than 0.05, and they need to be present in the model. Considering that the non-consuming code is 0 and the consuming code is 1, the probability of predicting social support is less than 1, so the social support variable is deemed effective.

**Table 8:** Variables in the equation

		B	S.E.	Wald	Df	Sig.	Exp (B)
**Step 1**	Social support	− 0.559	0.072	59.510	1	0.000^**^	0.572
	Self- control	0.028	0.143	0.038	1	0.845	1.028
	Constant	2.536	0.503	25.396	1	0.000^**^	12.627

**p<0.01

## Discussion

The present study aimed to compare social support and self-control among tobacco consumers and non-consumers in Hormozgan province. Results revealed that there is significant difference between tobacco consumers and non- consumers in terms of gender, education level, age, marital status and job status. This finding is consistent with findings reported by Hosseini et al. [25], Shuabi et al. [26], Yang et al. [27], Oura et al. [28], Tran et al. [29], Jamil et al. [30] and Amalia et al. [31]. The findings reported by Hosseini et al. [25] among the general population of Hormozgan province showed that there was a significant relationship between smoking status and gender, marital status, education and job status. Thus, the male, the married, the low literacy, the unemployed and the self-employed significantly consumed more tobacco than others. However, the female, those holding a diploma or a higher degree, employees or students were more non-consumers than others. The findings of a study conducted by Shuabi et al. [26] showed that women were less likely to smoke than men. Compared to the employed, those who were unemployed or retired were found to smoke more. The results of Yang et al.‘s study [27] showed that participants’ age, marital status, ethnicity, education, occupation, and average personal annual income were significantly associated with an increased likelihood of smoking among rural Chinese male residents. In a study conducted by Jamil et al. [30] significant risk indicators for smoking hookah were being younger than 22 years and living with a family member who used tobacco. The results of Amalia et al.‘s [31] study regarding changes in smoking patterns among Indonesian adults between 2007 and 2014 showed that in 2014, males, individuals under 55 years old and those with lower levels of education had a higher likelihood of being smokers. Those with a lower level of education and those under 26 years of age had higher odds of initiating smoking during the study period. Similarly, quitting smoking between 2007 and 2014 was more likely among respondents with a higher level of education and age above 40 years.

The results showed that the mean scores of social support, the support received from the family and important others in non-consumers was significantly higher than consumers. This finding was consistent with previous studies conducted by Poghosyan et al. [32], Romano et al. [33], Meijer et al. [34], TaraghiJah et al. [35], Marvizadeh et al. [36], Zaddahesh & Babakhani [37]. The results of Poghosyan et al.‘s [32] study in cancer survivors showed that survivors who received higher levels of social support were less likely to be current smokers than those who received the lowest level of social support they needed. The results of Romano et al.‘s [33] study among African-American adults in San Francisco and Oakland revealed that women with poor social networks smoked more than women with optimal networks. However, this relationship did not hold among men. Indeed, men lacking emotional support from friends or family were less likely to smoke than peers who received such support. In a study conducted by TaraghiJah et al. [35], they found that family emotional support was a strong predictor of hookah consumption and cigarette smoking among college students. Marvizadeh et al. [36] found that from the components of the relationship quality, only social support could predict students’ motivations (incremental, coping, and social) of cigarette and hookah smoking. In Meijer et al.‘s study [34], smokers from all socioeconomic levels were willing to receive positive social support if they would quit smoking. In Zaddahesh and Babakhani’s [37] study, the family support component was the strongest predictor of high-risk behaviors including drugs, smoking, and violence factors in adolescents. In explaining this finding, it can be stated that a lacking proper perception of social support from the family creates a feeling of emptiness and weakness, so individuals might show risky behaviors to fill the existing gap. Moreover, family challenges can lead to risky behaviors and insufficient individual and personal support [38]. In many cases, proper emotional relationships in the family can be considered as a major factor in preventing the occurrence of risky behaviors. Increasing the presence of parents at home and strengthening the emotional connections among family members not only ensures the family members’ psychological security, but also can be considered as an important factor in preventing addiction and its consequences [39].

And finally, the results revealed no significant difference between the mean scores of self-control, self-discipline and impulse control in consumers and non-consumers. This finding is inconsistent with findings of studies conducted by Bashirian et al. [40], Bazazian et al. [41], Ghadampour et al. [42], Agbaria et al. [43], Delalatgar et al. [44], Franken et al. [45], Allahverdipour et al. [46] and Daly et al. [20]. The results of the studies showed a significant relationship between self-control and high-risk behaviors [44], tendency to drug addiction [42, 43], tendency to cigarette smoking and alcohol consumption [40, 41, 43]. In Daly et al.‘s [20] study among Dutch adults, those with low self-control showed a large reduction in heavy smoking, while those with high self-control did not. The results of Franken et al.‘s study [45] showed that personal low self-control predicted an increase in externalizing behaviors (behaviors including antisocial behavior, alcohol consumption, and tobacco consumption) among adolescents. In the study of Allahverdipour et al. [46], students with poor self-control reported that they significantly consumed drugs and smoked cigarettes and were forced by their peers into consuming drugs and cigarettes. The inconsistency in findings can be attributed to different research populations and data collection methods (online, where some participants may have difficulty understanding some questions).

The first limitation of this study is that online surveys can be only used by some people, and those who do not have access to online surveys (e.g., the elderly, rural), people who do not have access to the Internet and people with low literacy, entered the study less than others. Second, the current research design was correlational and it did not seek to establish cause and effect, drawing any casual conclusions from the results is not possible. Third, since the present study only investigated adults, its result may not have been generalizable to other age groups. Therefore, it was suggested that future studies should be conducted to explore different age groups. It is recommended that future studies should be carried out to investigate other potential factors (such as personality traits, parenting styles, etc.) and more indicators of risky behaviors (such as drug abuse, alcohol consumption, risky sexual behaviors, violence, etc.).

## Conclusion

The present findings showed that tobacco consumers received less support from family and others compared to non-consumers. Based on these findings, designing and implementing psychological interventions and training useful skills to families, in order to increase social support from their children, can help to prevent and reduce the tendency to tobacco consumption. Theses interventions and trainings include: teaching appropriate communication patterns in family, teaching correct parenting or educational styles, creating an environment for a moral and logical discourse among family members, giving autonomy to a reasonable extent and correct control, unconditional acceptance of parents, etc.).

### Policy implications

It is recommended that the honorable officials of the health departments in universities of medical sciences plan for holding educational workshops and psychological interventions in health centers. Theses trainings should be planned and implemented with the aim of reducing the underlying factors of the tendency to tobacco consumption, including increasing the perceived social support.

## Supplementary Information

**Additional file 1.**

## Data Availability

The datasets generated and analyzed during the current study are not publicly available due to confidentiality and privacy related issues but are available from the corresponding author on reasonable request.

## Abbreviations

SD: Standard Deviation
