# Supplementary material for: The role of social support and self-control in tobacco consumption: a cross-sectional study among tobacco consumers and non-consumers
Source: BMC Psychol. 2023 Jun 29;11:192. doi: 10.1186/s40359-023-01226-y (PMC10311828; doi:10.1186/s40359-023-01226-y)
Supplement: Supplementary file 1 — Additional file 1. [file 40359_2023_1226_MOESM1_ESM.docx]

1. What is your gender?

Male Female

1. How old are you?

15-28 years old 29-39 years old 40-49 years old 50-80 years old

1. What is your level of education?

Under diploma Associate degree Bachelor's degree Master's degree and higher

1. What is your job?

University student Unemployed Housewife Employee Self-employed

1. What is your marital status?

Single Married
